# Solid Lipid Nanoparticles for Efficient Oral Delivery of Tyrosine Kinase Inhibitors: A Nano Targeted Cancer Drug Delivery

**DOI:** 10.34172/apb.2022.041

**Published:** 2021-07-03

**Authors:** Sukanta Satapathy, Chandra Sekhar Patro

**Affiliations:** Centurion University of Technology and Management, Odisha, India.

**Keywords:** Solid lipid Nanoparticles (SLNs), Tyrosine kinase inhibitors (TKIs), Bioavailability

## Abstract

Tyrosine kinase inhibitors (TKIs) are used as targeted therapy for cancer by inhibiting the signaling pathway and tumor growth. Many TKIs got approved by FDA in recent times for the treatment of cancer by oral route. However, the TKIs have formulation challenges leading to compromised bioavailability which can cause a weak therapeutic response. The cancer nanotherapeutics using nanocarriers based drug delivery has emerged as an advanced tool to provide a solution to formulation challenges and a better cancer therapy by overcoming the limitations in conventional cancer therapy. This review describes the various formulation issues of anticancer drugs with a special reference to TKIs, as well as the capability of solid lipid nanoparticles (SLNs) for an efficient nano targeted cancer drug delivery.

## Introduction

Cancer is a deadly disease posing a global threat to 185 countries with 19.2 million new cancer cases and 9.9 million deaths reported in 2020. GLOBOCAN 2020, supported by the International Agency for Research on Cancer, WHO, estimates cancer incidences and death for 36 types of cancers in 185 countries. This data is available in Global Cancer Observatory, an online web-based database.^1^ The future statistics revealed the possibility of a sharp rise of cancer new cases from 19.2 million in 2020 to 30.2 million in 2040.^2^ The treatment options available for cancer are surgery, radiation therapy, chemotherapy, immunotherapy, targeted therapy, hormone therapy, stem cell transplant, and precision medicine.^3^ The conventional chemotherapy drugs suffer from constraints such as, lack of bioavailability and poor aqueous solubility, tissue toxicity due to nonspecific biodistribution, lack of targeted drug action, instability in circulation, drug resistance, and limited cellular uptake. These limitations led to the lesser cytotoxic activity of anticancer drugs and ultimate sub-optimal therapeutic efficacy and patient cure.^4^ Targeted cancer thera­pies involve the blocking of the molecular targets with anticancer drugs specifically inhibiting the molecular target and thus, the growth and metastasis of the cancer are stopped. Targeted cancer therapy can solve the limitations of conventional chemotherapy, such as, nonspecific biodistribution and cell targeting. The various targeted therapies use small molecular drugs, monoclonal antibodies and signal transduction inhibitors.^5^ The tyrosine kinase inhibitors (TKIs) are nano-materials used to stop cell signaling by inhibiting signal transduction pathway. It has created a new hope in cancer treatment in the current age.^6^ However, the various formulation challenges of TKIs are to be addressed by formulation scientists to improve the efficacy of the existing drugs.^7^ The cancer nanotherapeutics using nanocarriers for drug delivery has emerged as an advanced tool to address the formulation challenges and better cancer therapy. The strategy can meet the limitations in the conventional cancer therapy with superior active and passive drug targeting.^8^ The nanocarriers such as, polymeric nanoparticles, micelles, dendrimers, liposomes, solid lipid nanoparticles (SLNs), nanotubes, loaded with chemotherapeutic drugs can be developed to improve the limitations of conventional anticancer treatment and develop highly capable anticancer drugs in terms of therapeutic activity and functionality.^8^ The various limitations of liposomes are limited drug loading capacity, drug leakage and vascular instability. On the other hand, the limitations of polymeric nanoparticles are nonavailability of large-scale production methods, polymer cost, cell toxicity of polymeric material, and toxic solvent residue. Presently, the lipid-based nano-carriers (SLNs etc) have drawn attention of researchers to deliver the active agents to the desired target with many advantages over the limitations of other nanocarriers.^9^ These solid lipid nanocarriers are also capable to address the formulation challenges associated with the TKIs to improve their bioavailability.^10^ Many researchers found that TKIs can be formulated into various nano-carrier-based drug delivery to improve their anticancer capacity. This review summarized the SLN formulation approach for delivery of the TKIs with enhanced anticancer potential.

## Tyrosine kinase inhibitors

Tyrosine kinase is the enzyme that helps in the phosphorylation of proteins and activates a signal transduction pathway leading to cell growth, differentiation, apoptosis and angiogenesis in a normal cell. Mutation in these tyrosine kinases leads to defective signal transduction and ultimately causes tumor growth.^11^ Tyrosine kinases can be classified as receptor protein kinases, which involve the transduction of extracellular signals into active intracellular signal transduction proteins, and nonreceptor protein kinases, which are involved in the transduction of signals within the cell.^12^ Receptor tyrosine kinases, such as epidermal growth factor receptor (EGFR) and vascular endothelial growth factor receptor (VEGFR), platelet-derived growth factor receptor (PDGFR) are involved in cancer proliferation and angiogenesis. Thus, inhibition of these receptors can stop the signaling pathways and ultimately inhibit the cancer growth and proliferation.^13^ The TKIs are used as a targeted cancer therapy, by inhibiting the signaling pathway and tumor growth. Many TKIs are approved by the FDA for cancer treatment. The updated list can be found at https://www.icoa.fr/pkidb/.^14^ Oral administration of chemotherapy has more advantages over the parenteral route. The major advantages are greater patient convenience and the flexibility of drug exposure.^15^ The bioavailability of the TKIs which are administered orally, is dependent on various gastrointestinal factors such as absorption and first-pass metabolism.^11^ Thus, their bioavailability is a major issue for an optimized formulation. The various factors that affect the bioavailability of TKIs are discussed to have a better approach to deal with the formulation issues, which can provide an efficient cancer therapy.

## Formulation issues of TKIs

The formulation of TKIs is a major challenge due to variable bioavailability. The various issues of formulation are discussed in the following sections.

## Poor oral bioavailability of TKIs

The small molecule TKIs (smTKIs) are used orally for targeted drug delivery in cancer chemotherapy. However, highly variable pharmacokinetics is observed with the smTKIs, which ultimately leads to poor oral bioavailability.^16^ Bioavailability is determine by the rate and extent of drug absorbed into the systemic circulation. The poor and variable bioavailability of smTKIs may result in variable plasma level, which can lead to decreased therapeutic response.^7^ Table 1 presents the BCS classification of different TKIs. Various factors, either single or in combination are responsible for the poor bioavailability of the TKIs. The physicochemical, biological and other factors that influence the bioavailability of smTKIs are discussed.^17^ The bioavailability of various TKIs are given in the Table 2.^18,19^ Apart from variable bioavailability, the inter individual variation in pharmacokinetics can affect the dose response, either overdose or underdose, leading to toxicity or development of resistant clones.^16^ Therapeutic drug monitoring is an essential approach for TKIs for dose adjustment to have a better response and reduced side effect due to fixed dose for every indivisual.^11,20^ The interplay of various factors, for a poor and variable bioavailability are discussed below.

**Table 1:** BCS classification of some tyrosine kinase inhibitors

**BCS Class**	**Solubility**	**Permeability**	**TKIs**
I	High	High	Afatinib
II	Low	High	Axitinib, Carbozantinib, Dabrafenib, Dasatinib, Erlotinib, Gefitinib, Lapatinib Pazopanib, Regorafenib, Vandetanib
III	High	Low	Afatinib
IV	Low	low	Bosutinib, Crizotinib, Nilotinib Sunitinib, Vemurafenib

**Table 2:** Physicochemical properties and bioavailability of some TKIs

**Name of TKIs**	**MW** ^a^ ** (Da)**	**Bioavailability (%)** ^b^	**Solubility** ^c^ ** mg/mL**	**log p** ^c^	**Primary target** ^d^	**IC50 in nM** ^e^
Afatinib	486	**-**	0.0128	3.77	ErbB1/2/4	10,14,1
Alectinib	483	37	0.0105	5.59	ALK, RET	1.9
Avapritinib	499	-	0.0301	2.68	PDGFR	0.5
Axitinib	386	58	0.000551	4.17	VEGFR1/2/3	0.1,0.2,01-0.3
Brigatinib	584	-	0.022	5.11	ALK	0.6
Cabozantinib	501	-	0.00199	4.01	VEGFR2, RET	0.035,4
Capmatinib	412	-	0.00529	3.04	c-MET	0.13
Ceritinib	558	25	0.00222	5.23	ALK	0.2
Crizotinib	450	43	0.00611	3.82	ALK, ROS1	24,<0.025
Dacomitinib	470	80	0.00874	4.88	EGFR	6
Entrectinib	561	-	0.0089	5.03	TRKA/B/C, ROS1	0.1 to 1.7
Erdafitinib	446	-	0.013	3.57	FGFR1/2/3/4	----
Erlotinib	393	60	0.00891	3.13	EGFR	2
Fostamatinib	580	55	0.052	2.78	Syk	41
Gefitinib	447	60	0.027	4.02	EGFR	26
Gilteritinib	552	-	0.0223	3.51	Flt3	0.29
Lapatinib	580	<25	0.0223	5.18	ErbB1/2/HER2	10.8,9.2
Larotrectinib	428	34	0.238	2.07	TRKA/B/C	----
Lenvatinib	427	-	0.00622	3.03	VEGFR1, RET	22
Lorlatinib	406	81	0.108	2.01	ALK	--
Midostaurin	571	-	0.0157	4.52	Flt3	912(FLT1)
Neratinib	557	-	0.00674	4.72	ErbB2/HER2	59
Nintedanib	540	5	0.0309	3.7	FGFR1/2/3	69,37,108
Osimertinib	500	-	0.0224	4.47	EGFR T970M	11.44
Pazopanib	438	14-39	0.0433	3.59	VEGFR1/2/3	10,30,47
Pemigatinib	487	-	0.144	2.26	FGFR1/2/3/4	0.4,0.5,1.2,30
Pexidartinib	417	-	0.00315	4.64	CSF1R	20
Pralsetinib	534	-	0.0101	3.63	RET	0.3
Regorafenib	483	69-83	0.00102	4.53	VEGFR1/2/3	13,4.2,46
Ripretinib	510	-	0.00583	4.3	KIT/PDGFR	4
Selpercatinib	526	73	0.0299	3.03	RET	1
Sorafenib	465	-	0.00171	4.12	VEGFR1/2/3	15,90,20
Sunitinib	398	-	0.0308	3.24	VEGFR2	80
Tucatinib	481	-	0.004	3.87	ErbB2/HER2	8,7
Upadacitinib	380	-	0.0707	2.57	PDGFR	-
Vandetanib	475	-	0.0102	5.01	VEGFR2	40

Abbreviations: EGFR (epidermal growth factor receptor), HER (human epidermal growth factor receptor), PDGFR (platelet derived growth factor receptor), VEGFR (vascular endothelial growth factor receptor), FGFR (fibroblast growth factor receptor), RET (rearranged during transfection), ALK (anaplastic lymphoma kinase), CSF (colony stimulating factor). ^a^Data taken data from NIH PubChem. ^b^ Available data from registration documents by FDA.^c^ Data taken from Drug Bank. ^d^ Data taken from Blue ridge institute for medical research.^51e^ Data taken from selleckchem.^52^

Factors affecting bioavailability of TKIs are

- Physicochemical factors:Drug aqueous solubility and dissolution, drug degradation and stability in the gastrointestinal tract, lipophilicity of the drug, size of the drug molecule
- Food and drug interaction factors:Food effect, drug interaction with acid reducing agents
- Biological barriers /Physiological factors:Trans membrane efflux of the drugs (P-gp efflux pump), first pass metabolism (intestinal and liver cytochrome P450 metabolic enzymes).

## Physicochemical factors

### Drug solubility and dissolution

The solubility enhancement is essential for BCS class II drugs. The bioavailability of these classes of drugs are solubility/dissolution rate limited, but not limited by the permeability rate.^21^ The BCS (Biopharmaceutics classification system) class is assigned to the drugs as per their water solubility and GI membrane permeability characteristics.^22^ The assigned BCS class data are taken from the FDA clinical pharmacology, biopharmaceutics review documents and the published reviews.^23,24^ The various BCS classes assigned to different TKIs are represented in Table 1. The increase in Bioavailability of BCS class II drugs can be achieved by enhancing the dissolution.^21^ The TKIs exhibit poor solubility (listed in the Table 2) and thus, there is a need for solubility enhancement to have good oral bioavailability. The TKIs are given orally. Dissolution is the initial step for oral absorption. The GI fluid solubility of the drug is necessary for the oral absorption of drugs. The TKIs are weakly basic and they show pH-dependent aqueous solubility. GI pH is an important factor that affects absorption and bioavailability. The GI pH profile includes stomach with a highly acidic pH 2, Jejunum with slightly acidic pH 5-6, and Ileum with slightly alkaline pH 7-8.^25^ The TKIs are absorbed mostly from the small intestine which has a larger surface area due to the epithelial folding and villous structure. The drug solubility of TKIs in the small intestine is needed for absorption. The small intestine transit time for the drugs is observed to be 3-4 hours and the value does not change with the presence of food.^26^ The TKIs are weak bases; hence, upon oral administration when they reach stomach and due to acidic pH in the stomach they get ionized. As a result, the drug solubility increases in the stomach, but in the intestine, due to the increase in pH, the solubility decreases. Many of the TKIs show the pH dependent solubility.^27^ The solubility of TKIs affects the design of a good formulation.

### Drug degradation and stability in the GI tract

The stability of drugs is affected by acidic pH and ultimately affects absorption and bioavailability. The chemical stability of a drug in the GI tract is affected by the pH.^28^ The anticancer drug etoposide and chlorambucil show poor chemical stability in GI fluids leading to variable and low bioavailability.^29^ The SLNs provide matrix encapsulation of the drug which leads to protection from acid instability and also helps in sustained release. Drug metabolism by hydrolysis in GIT and plasma is also protected by the SLN carrier drug delivery.^30^ Thus, the TKIs can provide significant advantages in the SLN formulation.

### Lipophilicity of the drug

Lipophilicity determines the passive permeability of the drugs. The log *P* values (representing the partition of the drug between octanol and aqueous buffer at a pH of 7.4 as a measure of lipid solubility) are the measure of lipophilicity. If the log *P* value is below –0.4, the compound faces difficulty to cross the GI barrier. However, this will be facilitated if the log *P* value is above 5.^31^ The uncharged fraction of the drugs can cross the GI membrane while the charged fraction cannot. For weak acids and bases (weak base like TKIs) the pka values are considered for the membrane permeability because the pka represents that value of pH at which 50% of the drug is in ionized form and 50% of drug exists in the unionized form.^17^ Lipinski’s rule of five specifies states that the partition coefficient should not be more than five^32^ and the values in the range 1-3 show good absorption and values of log *P* < 1 and > 3 show poor penetration. Partition coefficients of the drugs in the range of 1–3 show good passive absorption across intestinal barriers and log *P* values outside the limit (1 and 3) have poor transportation characteristics.^33^ The log *P* values of the various TKIs are given in Table 2. The lipophilicity of TKI is an important parameter that affects bioavailability.

### Size of drug and bioavailability

The high molecular weight of the drugs can cause difficulty to cross the GI membrane by passive diffusion. The molecular weight, lipophilicity and surface polarity determine the membrane permeation of the drug.^34^ Lipinski rule also states that the molecular weight, if greater than 500 Da, leads to large size of the molecule, hampers passive absorption due to concentration gradient, and leads to low bioavailability due to the slow absorption.^32^ The molecular weights of various TKIs are given in Table 2.The size of the TKIs affects bioavailability.

## Food and drug interaction factors

### Food effect with TKIs

The interaction of food with the administered drug also affects bioavailability. The buffering effect, dilutant effect of food, the composition of GI fluid during feed state resulting in the change in pH of the gastric environment, and change in solubility of drugs lead to less dissolution. Food can affect gastric emptying rate. Change in residence time, modulate efflux transporter and metabolizing enzymes can also affect the absorption of drugs and bioavailability.^35^ The TKIs are oral targeted drugs with positive food effects, which means the administration of TKIs with food increases the absorption.^36^ The TKIs taken with food may cause toxicity due to increased concentrationof anticancer drugs. Hence these drugs are not taken with food. These drugs are taken before 2 hours or after one hour of taking food.^36^ Lipid formulations such as, SLNs resemble the high fat content of a meal which increases solubilization of the administered drug molecule.^37^ TKIs show the variability in the absorption and bioavailability in presence of food.^38,39^ The grapefruit juice contains furanocoumarins (bergamottin, 67-dihydroxybergamottin)which irreversibly inhibit the intestinal metabolism, and the plasma concentration of the drugs gets increased leading to adverse effect for TKIs.^40^

### pH regulating drugs and TKI interaction

The TKIs are weak bases and show pH dependent solubility. The concurrent administration with acid regulating drugs like Proton pump inhibitors, antacids, and H_2_ receptor antagonists can raise the pH so that the solubility and absorption are affected.^27,41^ Hence TKIs are to be administered as per the drug interaction prescribing information. The concentration of dasatinib decreases with simultaneous administration of proton pump inhibitors or H_2_ antagonists due to the increase in pH and decrease in solubility of the drug, which results in the reduced efficacy of dasatinib.^42^

## Biological barriers/Physiological factors

### Efflux transporter proteins of GI barrier and multidrug resistance

The physiological drug barrier is the GI membrane. The membrane transporter proteins are the molecular cause of impermeability of various anticancer drugs.^29^ The various membrane drug transporters such as, ATP binding cassette transporters (ABC transporter) like permeability-glycoprotein (P-gp), the multidrug resistance-associated proteins, and the breast cancer resistance protein (BCRP) are found to impact the absorption of anticancer drugs by acting as an efflux pump limiting the drug permeability and bioavailability for TKIs.^7^ The TKIs act as substrate or inhibitors of ABC transporters and these ABC transporters are involved in active drug efflux which can cause drug resistance for the TKIs.^43^ TKIs are taken up into the cells by the Solute carrier transporter or SLC transporters.^44^ The various organic anion transporter proteins and organic cation transporter proteins are a subfamily of SLC inhibitors and the TKIs may act as substrate or inhibitors for the SLC inhibitors and found to have interaction influencing the absorption of TKIs.^43^ The lysosomal sequestration of TKIs is also a cause of MDR in TKIs.^43^ All these efflux can lead to the variable bioavailability of the TKIs.

### First-pass metabolism and bioavailability

The site for the first-pass metabolism of the drugs is the intestine and liver. The enzymes in the intestine and liver are responsible for first-pass metabolism. Cytochrome P450 (CYP) enzymes (phase I metabolism) and other conjugating enzymes (phase II metabolism) are the enzymes for the metabolism of the drugs in the liver. The enzyme CYP3A4 is the metabolizing enzyme in the intestine.^45^ CYP3A4 is the most important phase-1drug-metabolizing enzyme in the body and is the mostly found as the isoform of the enzyme in the liver.^46^ The drugs are metabolized by the enzymes before absorption in the intestine and after absorption, the drugs enter into the enterohepatic circulation to be metabolized by the enzymes in the liver leading to the low bioavailability of drugs. The metabolized drug may act as a substrate for the Pgp and this concept of interactive action is also a highly emerging factor for interactively reducing the bioavailability.^47^ The role of various metabolizing enzymes such as, uridine diphospho-glucuronosyltransferases (UGTs), glutathione-S-transferases (GSTs), dihydropyrimidine dehydrogenases, and thiopurine methyltransferases, for creating drug resistance are also studied.^48^ Most of the TKIs are metabolized by CYP enzyme, CYP3A4, and also glucuronidation by UGTs,^49^ which may account for low bioavailability. TKIs are found to be the substrates of CYP3A4; hence, the CYP3A4 Inhibitors or inducers can change the bioavailability of the TKIs. The simultaneous use of these inhibitors or inducers with TKIs cannot be overlooked. The area under the curve of sunitinib and nilotinib increased by 11% and 29%, respectively, with grapefruit juice which is an inhibitor of CYP3A4.^50^ Thus, the First-pass metabolism affects the bioavailability of TKIs.

## Solid lipid nanoparticles

SLNs are colloidal nano drug carriers with particle size ranging between 50 and 1000 nm.^53^ SLNs are made up of solid biodegradable lipids as a solid matrix core covered by hydrophilic surfactant.^54^

## Advantages of SLNs

The SLNs have excellent biocompatibility and low toxicity. The lipophilic drugs are better delivered by SLNs.^55^

The SLNs are made from physiologically compatible lipids. SLNs also show less cytotoxicity as compared to the polymeric nanoparticles.^56^

Feasibility of large scale production, high product stability, biodegradability, increased entrapment efficiency, controlled drug release, drug targeting by surface modification are the advantages of SLNs over the liposomes and polymeric nanoparticles.^57^

SLNs also show controlled drug release by the degradation, erosion, or diffusion of the lipid matrix.^53^

The SLNs, can deliver the TKIs, with enhanced bioavailability and decreased resistance. Combination drug delivery with targeted therapy also possible.^10^ Lipid based nano carriers such as, SLNs can improve the limitations of conventional anticancer treatment by highly capable anticancer drugs in terms of therapeutic activity and functionality.^53^

## Formulation of SLNs

General formulation ingredients include solid lipid(s), emulsifier(s) with API (drugs, proteins). The other ingredients used are co-surfactants, preservatives, cryoprotectants, and charge modifiers. The lipids used are triglycerides (e.g. tristearin), partial glycerides (e.g. Imwitor), fatty acids (e.g. stearic acid), steroids (e.g. cholesterol), and waxes (e.g. cetyl palmitate). All classes of emulsifiers, which includes various surfactants (tweens), organic salts are used to stabilize the lipid dispersion.^58^ The detailed ingredients generally used to prepare SLNs are given in Table 3.The ingredients used to prepare TKI SLNs are specifically cited in the reference column of Table 3.

**Table 3:** List of ingredients used for the preparation of SLNs^53,58^

**Lipids**	**References**	**Surfactants/Emulsifiers**	**References**
**Triglycerides**		**Phospholipids**	
Tricaprin		soybean lecithin (Lipoid S 75, Lipoid S 100)	^59,60^
Trilaurin		Egg lecithin (Lipoid E 80)	
Trimyristin (Dynasan 114)		**Ethylene oxide/propylene oxide copolymers**	
Tripalmitin (Dynasan 116)		Phosphatidylcholine (Epikuron 170, Epikuron 200	
Tristearin (Dynasan 118)		Polaxamers 182	
Hydrogenated coco-glycerides (Softisan 142)		Polaxamer 188 (PLURONIC F-68)	^59,61^
**Hard fat types**		Polaxamer 407 (PLURONIC F-127)	^62,63^
Witepsol W35		Poloxamine 908	
Witepsol H35		Tyloxapol	
Witepsol H42		**Sorbitan ethylene oxide/propylene oxide copolymers**	
Witepsol E85		Polysorbate 20,60,80	^62^
**Acyl glycerols**		**Bile salts**	
Glyceryl monostearate (GMS) (Imwitor 900)	61	Sodium cholate	
Glyceryl Behenate (Compritol 888 ATO)	59,62	Sodium glycocholate	
Glyceryl palmitostearate (Precirol ATO 5)		Taurocholic acid sodium salt	
**Waxes**		Taurodeoxycholic acid sodium salt	
Cetyl Palmitate (Crodamol CP)		**Alcohol**	
**Fatty acids**		Butanol	
Stearic acid	60,63	Butyric acid	
Palmitic acid		Dioctyl sodium sulfosuccinate	
Decanoic acid		Monooctylphosphoric acid sodium	
Behenic acid			
Acidan N12 (monostearate monocitrate diglyceride)			

## Methods of preparation of SLNs

The various methods of preparation of SLNs include high shear homogenization, ultrasonication or high speed homogenization,^59^ high pressure homogenization (cold homogenization, hot homogenization^62^), microemulsion based methods, supercritical fluid method, solvent emulsification method,^60^ solvent evaporation method,^60,61^ double emulsion methods, precipitation techniques and spray drying methods.^53,64^ The various TKIs formulated into SLNs by using various methods are given in Table 4.

**Table 4:** Method of preparation of SLNs formulation of TKIs.

**TKI (SLN formulation)**	**Method of preparation**	**Reference**
Erlotinib loaded with SLN and formulated as a Dry powder inhaler.	hot homogenization method	^62^
Gefitinib SLNs as a dry powder inhaler	emulsion-solvent diffusion and evaporation method	^63^
Sorafenib SLNs for oral administration	high-speed shearing and ultrasonic treatment	^59^
Ceritinib SLN	Single emulsification and solvent evaporation	^61^
Brigatinib SLN	solvent emulsification/evaporation technique using probe-sonication.	^60^

## Route of administration and Applications of SLN

SLNs can be administered by various routes such as, oral, parenteral, nasal, topical or transdermal, ocular, rectal for various types of drugs.^65,66^ The SLNs find their applications in medicine, food science, cosmetics, dermaceuticals, and phyto pharmaceuticals nanotherapeutics for efficient delivery.^67^ Cancer chemotherapy finds its efficient and safe delivery by using SLNs as a nanocarrier.^68^ SLNs can incorporate a number of anticancer drugs and have proven to be effective in different types of tumors at breast, lung, colon, liver and brain.^69^ Table 5 summarizes various outcomes of TKIs through SLN delivery system.

**Table 5:** Efficient outcomes of TKIs through SLN delivery system

**TKI with SLN delivery**	**Outcome**	**Reference**
Erlotinib loaded with SLN and formulated as a dry powder inhaler	Encapsulation efficiency is 78.21%, erlotinib-SLNs show enhanced cytotoxicity.	^62^
Gefitinib SLNs as a dry powder inhaler	The encapsulation efficiency of 97.31 ± 0.23 %, superior anticancer effect as compared with free gefitinib.	^63^
Sorafenib SLNs for oral administration	Drug selectivity index value which measures the liver targeting of sorafenib-SLNs was 2.20 times higher and AUC increased by 66.7% than that of the sorafenib suspension.	^59^
Ceritinib	The in vitro studies indicate a maximum drug release of 95.12% in 360 min as compared to (30.12% in 360 min). Stability is more even after 90 days.	^61^
Brigatinib	The optimized formulation is more cytotoxic with 74.91% less dose as compared with the brigatinib suspension. Entrapment efficiency is 87.09 ± 0.68% and drug loading is 7.86 ± 0.44%.	^60^

## SLNs for efficient delivery of TKIs

The oral delivery of TKIs is a challenge for the formulation researchers due to various formulation issues as discussed earlier. These issues can be addressed by using SLN as a carrier for drug delivery in an efficient manner.

## SLNs for improving solubility for TKIs

SLNs are a new formulation strategy for improving the bioavailability of various poorly water-soluble drugs. SLNs were found to improve the absorption of solubility hindered drugs.^70^ The researchers use various techniques for improving the solubility of poorly water-soluble drugs include micronization, nanonization, nanoemulsion, prodrug, salt formation, co-crystallization, chemical modification, polymorphs, pH adjustment, solid dispersion, complexation, co-solvency, micellar solubilization, polymeric micelle, hydrotropy, self-emulsifying drug delivery system (SEDDS), liposomes, niosomes, SLNs, nanostructured lipid carriers, etc.^21,71^ For the TKIs which belong to class II and IV, the enhancement of bioavailability can be achieved through dissolution improvement with different strategies such as, particle size reduction, self-emulsification, cyclodextrin complexation, crystal modification, and amorphous solid dispersion. The targeted drug delivery for specific biodistribution to reduce side effects and improved therapeutic efficacy along with bioavailability improvement can be achieved by dendrimers, polymeric nanoparticles, magnetic nanoparticles, and lipid based delivery systems such as liposomes, SLNs, and nanostructured lipid carriers (NLCs).^10^ The conventional molecular optimization to improve the pharmacokinetics has been seen to be improved with nanoformulation approaches to deal with the various issues of bioavailability.^33^ The various polymeric nanocarriers, for example, polymeric nanoparticles, polymeric micelles, polymer drug conjugate, and lipid based nanocarriers are used for drug delivery through Emulsions, SEDDS, NLCs, lipid nanocapsules, layersomes, lipid drug conjugates, SLNs. They are found to be good drug delivery systems for anticancer drugs.^72^ Lipid formulations can improve solubilization, absorption, and minimize the food effect.^37^ Thus, ultimately the bioavailability of TKIs is improved.

## Nano size of SLNs to enhance the bioavailability of TKIs

The SLNs used for the drug delivery carriers are of small size of 50-1000 nm and the absorption increases due to the increase in the surface area. This small size also favors bypassing the physiological barrier of the GI tract. In this way, the SLNs can improve the bioavailability of the small molecule TKIs.^73^

## SLNs for reversing multidrug resistance of TKIs

The SLNs are a better approach to reduce or reverse the multidrug resistance. The TKIs are found to inhibit the ABC transporters. A combinational strategy of treatment with other anticancer drugs can be formulated to reduce the anticancer drug resistance.^74^ However, P-gp inhibitors used to reduce drug efflux by membrane transporters, can cause complications by suppressing the immune system. Nanocarriers such as, SLNs are found to be a better approach to by-pass the efflux pump transport.^75^ The conventional anti MDR strategy has the limitations of pharmacokinetic interaction between combination drugs of P-gp inhibitor and chemotherapeutics, suppression of immunity and physicochemical formulation issues. The novel nano drug delivery strategy can utilize SLNs which can deal with the MDR by increasing the drug uptake into tumor cells, drug accumulation in tumor cells, suppressing MDR proteins such as P-gp, increasing the bioavailability of drugs, and inducing apoptosis.^76^ The recognition of nanoparticles by P-gp is avoided with the nanoparticles and these nanoparticles accumulate in the cells, which can improve the absorption.^77^ The cytotoxicity of SLN loaded anticancer drugs such as, paclitaxel and doxorubicin was found to be increased by reversing the resistance by multi drug resistant cancer cells.^78^ The SLNs can also be formulated for TKIs to reverse the MDR of TKIs.

## SLNs for bypassing first-pass metabolism of TKIs

The bioavailability can be improved with the simultaneous delivery of inhibitors of the ABC transporters and CYP450, however the inhibition of metabolizing enzymes may affect the bioavailability of xenobiotics and cause other side effects.^79^ The SLNs form the chylomicrons by enterocytes, thereby the lymphatic transport of lipophilic drugs is enhanced which results in bypassing the intestinal and hepatic metabolism and the bioavailability is improved.^80^ This approach can be used to increase the bioavailability of the TKIs which are the substrates for the metabolizing enzymes.

## PEGylated SLNs for efficient delivery of TKIs

The absorption barrier of the intestinal mucosa and mucus clearance of drugs are also causes of low bioavailability. Polyethylene glycol was introduced for coating lipid-based drug carriers to have a hydrophilic layer resulting in increased oral delivery of drugs against the viscoelastic mucus layers.^81^ The PEGylated SLNs (pSLNs) are prepared to evaluate the mucus penetrating capacity and found that the pSLNs can easily penetrate the mucosal barrier as compared to the SLNs. Thus, the absorption efficiency and blood circulation time increased with an increase in relative bioavailability of 1.99 times as compared to that of the SLNs.^82^ Based on the above discussion, it can be expected that the SLNs pegylation can improve the oral bioavailability of the various TKIs, which can be a point of research in the future formulation investigation.

## SLNs for lymphatic drug uptake to improve bioavailability

Intestinal lymphatic transport for lipids, either as food or lipophilic drug, is an alternate absorption route that opens a door for a lipid-based drug delivery system. The absorption of lipophilic drugs increases with co-administration with food of lipidic content. This concept provides the lipid based formulation approaches for drug delivery through the lymphatic route.^83^ Drugs with poor and variable oral bioavailability due to low solubility in the GI tract or pre-systemic hepatic metabolism, can be improved in terms of bioavailability by formulating into an SLN for lymphatic drug delivery with bypassing first-pass metabolism.^84^ The paracellular absorption, M cell uptake via Peyer’s patches, chylomicron-assisted enterocytes absorption are the different routes of drug absorption for SLNs.^30^ The lipid core of SLNs facilitates lipase mediated chylomicron formation through lipid digestion and performs lymphatic uptake by lymphatic transport and lymphatic route, which can bypass hepatic first-pass drug metabolism to improve the absorption and oral bioavailability of water insoluble drugs.^85^

## SLN reducing drug interactions with TKIs

The TKIs are extensively used in cancer therapy. So there is a possibility of drug interactions that may lead to additive QT prolongation and decreased TKI exposure.^41^ In oral cancer therapy, the cytotoxic effect to the GI tract is unavoidable, drug–drug interactions are observed in 46% of patients having oral cancer therapy treatment, out of which 16% were considered major interactions. This cytotoxic effect and drug interaction related side effect can be overcome with the nanoformulations.^72^

## SLN for increasing encapsulation efficiency of TKIs

Encapsulation efficiency and drug loading capacity are the two important parameters for the SLNs which determine the amount of drug associated with the nanoparticle or percentage of drug encapsulated into SLN, which in turn, determines the therapeutic efficacy for a drug.^86^ Erlotinib-loaded SLN based formulation of dry powder inhaler was prepared by Bakhtiary et al and it was found that the encapsulation efficiency was 78.21%.^62^ Satari et al^63^ prepared glucosamine conjugated gefitinib SLNs and the optimized formulation had drug loading of 33.29 %, encapsulation efficiency of 97.31 ± 0.23 %. Improved anticancer effect of gefitinib loaded SLN, as compared to that of free gefitinib was studied. Thus, the SLNs can be an efficient drug carrier for TKIs to improve the encapsulation efficiency.

## IC_50_ value and enhanced cytotoxic effect of TKIs with SLNs

The cytotoxic effect of a drug in a cancer cell is popularly studied by MTT assay, which is a cell viability test. An MTT assay is a simple and effective invitro assay for the quantification of cell viability and proliferation. A549 Lung adenocarcinoma cell line was used to see the cytotoxic potential of an anticancer drug.^87^ The drug concentration that reduces the viability of cells by 50% is termed IC_50_. The IC_50_ was extrapolated from the dose-response graph. Ahmed et al^60^ developed Brigatinib (BG) loaded SLNs by using solvent emulsification technique, characterized and MTT assay was performed on the optimized SLNs (BS5). It was found that the BG loaded SLN (BS5) showed better cytotoxicity against A349 lung cell lines while compared to BG suspension and blank SLN. The IC50 (µg/mL) values for blank-SLN and BS5 were found to be 89.9 ± 2.4 and 43.85 ± 1.8 respectively, however, IC50 for pure drug-BG was reported to be 58.53 ± 1.3 µg/mL. Therefore, it was concluded that optimized BS5 formulation could be relatively more cytotoxic, effective in 74.91 % less dose as compared to that of the drug-suspension (BG). These values can support the fact that SLN delivery of TKIs can deliver the drugs with enhanced cytotoxic effect.

## Sustained drug release of TKIs from SLNs

Drug release from SLNs follows the zero order diffusion controlled mechanism or erosion or degradation of the lipid matrix system of the SLN and a controlled release of the drug is obtained. An initial burst release or rapid release of drug is seen and is due to the weakly bound surface drugs on the SLN.^88^ The controlled drug release can be obtained for TKI inhibitors also. Imatinib (IMT) loaded SLNs formulations optimized with Plackett-Burman design and Box-Behnken design with variables like organic-to- aqueous phase ratio, drug-to-lipid ratio, and amount of Tween® 20 for particle size, drug loading, and encapsulation efficiency of IMT -SLN show the results with sustained release pattern of the drug with enhanced physicochemical characteristics.^89^ A sustainedrelease pattern was observed with the in-vitro release profile for brigatinib. Brigatinib loaded SLNs can find an important place in the non-small cell lung cancer treatment.^60^

## Conclusion

TKIs are a breakthrough in cancer targeted drug delivery. The nanoformulation with SLNs is a novel cargo for the TKIs. This approach has proven to be most efficient and the results also showed promising for TKIs. The future potential for TKIs loaded SLNs will be a more efficacious discovery for formulation scientists. Future cancer therapy with the reviewed approach with SLNs and loaded TKIs will be significantly beneficial for cancer treatment.

## Conflict of Interest

There is no conflict of interest.

## Ethical Issues

Not applicable.
